# The Combination of *β*-Asarone and Icariin Inhibits Amyloid-*β* and Reverses Cognitive Deficits by Promoting Mitophagy in Models of Alzheimer's Disease

**DOI:** 10.1155/2021/7158444

**Published:** 2021-11-30

**Authors:** Nanbu Wang, Haoyu Wang, Qi Pan, Jian Kang, Ziwen Liang, Ronghua Zhang

**Affiliations:** ^1^The First Affiliated Hospital, Guangzhou University of Chinese Medicine, Guangzhou, China; ^2^The First Affiliated Hospital, Jinan University, Guangzhou, China; ^3^College of Traditional Chinese Medicine, Jinan University, Guangzhou, China; ^4^Guangzhou University of Chinese Medicine, Guangzhou, China; ^5^College of Pharmacy, Jinan University, Guangzhou, China

## Abstract

*β*-Asarone is the main constituent of *Acorus tatarinowii Schott* and exhibits important effects in diseases such as neurodegenerative and neurovascular diseases. Icariin (ICA) is a major active ingredient of Epimedium that has attracted increasing attention because of its unique pharmacological effects in degenerative disease. In this paper, we primarily explored the effects of the combination of *β*-asarone and ICA in clearing noxious proteins and reversing cognitive deficits. The accumulation of damaged mitochondria and mitophagy are hallmarks of aging and age-related neurodegeneration, including Alzheimer's disease (AD). Here, we provide evidence that autophagy/mitophagy is impaired in the hippocampus of APP/PS1 mice and in A*β*1-42-induced PC12 cell models. Enhanced mitophagic activity has been reported to promote A*β* and tau clearance in in vitro and in vivo models. Meanwhile, there is growing evidence that treatment of AD should be preceded by intervention before the formation of pathological products. The efficacy of the combination therapy was better than that of the individual therapies applied separately. Then, we found that the combination therapy also inhibited cell and mitochondrial damage by inducing autophagy/mitophagy. These findings suggest that impaired removal of defective mitochondria is a pivotal event in AD pathogenesis, and that combination treatment with mitophagy inducers represents a potential strategy for therapeutic intervention.

## 1. Introduction

*β*-Asarone (1,2,4-trimethoxy-5-[(Z)-prop-1-enyl]benzene) is an essential component of *Acorus tatarinowii Schott* volatile oil that can easily pass through the blood-brain barrier. Our group has been working on *β*-asarone for nearly 30 years, and we found that it has a very good pharmacodynamic effect in AD. This monomer improved the behavioural symptoms of Alzheimer's rats and improved learning and memory abilities by inhibiting the PI3K/Akt/mTOR pathway [1–3]. Icariin (ICA) is a flavonoid constituent isolated from Epimedium that has been extensively researched in the field of regenerative medicine [4, 5]. In our previous research, we treated A*β*42-induced PC12 cells with Icariin and *β*-asarone at gradient concentrations. By exploring the effects of drug concentration and combination treatment, we found that the combination treatment had better therapeutic outcomes than the individual treatments [6]. AD is a complex disease, and intervention from a single mechanism of action may not be the optimal principle of drug therapy. Just like a blood pressure medication that combines calcium channel blockers with diuretics.

Dementia describes an intraindividual pattern of decline in memory and thinking that impairs at least two domains of cognition [7]. Alzheimer's disease (AD) is an irreversible, age-associated neurodegenerative disease that is characterized by progressive memory loss and cognitive decline [8]. The presence of extracellular senile plaques comprising A*β* (amyloid beta) is one of the main neuropathological hallmarks of AD [9, 10]. Despite the genetic evidence and the demonstrated involvement of A*β* in inducing synaptic dysfunction, which disrupts neural connectivity and is associated with neuronal death in a brain region-specific manner, the amounts and distribution of A*β* deposits are only weakly correlated with the clinical expression of the disease [11].

Autophagy is the main cellular mechanism for degrading and recycling intracellular proteins and organelles under different physiological and pathological conditions [12]. Mitophagy is a form of selective autophagy during which mitochondria are decorated with polyubiquitin chains, engulfed by autophagosomes, and degraded following lysosomal fusion [13]. This process involves the targeting of damaged or superfluous mitochondria to lysosomes, where mitochondrial constituents are degraded and recycled. In mammals, PTEN-induced kinase 1 (PINK1) and Parkin are necessary proteins for mitophagy [14].

The mitochondrial membrane potential (*ΔΨ*m) results from redox transformations associated with the activity of the Krebs cycle and serves as an intermediate form of energy storage that is used by ATP synthase to make ATP. These transformations generate not only an electrical potential (because of charge separation) but also a proton gradient, and together, they produce the transmembrane potential [15, 16]. Ca^2+^ is an important molecule that affects a wide range of physiological and pathological processes. Unlike other secondary messengers, Ca^2+^ is not synthesized or metabolized but is stored and rapidly released by channels/pumps that maintain Ca^2+^ concentrations in distinct cellular compartments [17]. The calcium hypothesis also reformulates one of the important theories about AD and offers evidence for a role of Ca^2+^ in AD pathogenesis [18]. Importantly, mitochondrial quality control plays critical roles in neuronal function and neuronal survival through the maintenance of a healthy mitochondrial pool and the inhibition of neuronal death [19, 20].

Our previous analysis provided evidence that defects in autophagy have a critical role in AD development and progression. The autophagy-lysosome pathway is believed to be another major A*β* clearance route in addition to the different A*β*-degrading enzymes [21]. Thus, targeting the maintenance of autophagy at both the neuronal and organismal levels may be a promising therapeutic approach. However, the role of mitophagy in AD progression is unclear.

This is the first time we have used the combination of *β*-asarone and ICA to investigate AD. Based on the evidence cited above, we detected changes in mitochondria and mitophagy in the APP/PS1 mouse and PC12 cell models and showed defective mitophagy in AD. Then, we analysed the pharmacological effect of the combination therapy using behavioural tests and measuring the levels of AD-related proteins. Furthermore, we used antimycin A (A3) and cyclosporine A (CSA) with the combination treatment as an activator and an inhibitor of mitophagy, respectively, to investigate the mechanism. To the best of our knowledge, our data demonstrate that this combination can protect against damage from toxic pathology and mitochondrial disruption and that this process occurs via promotion of mitophagy, which could act as a potential key contributor to therapeutic action.

## 2. Materials and Methods

### 2.1. Chemicals and Reagents

The A*β*1-42 used in these experiments was acquired from Life Technologies (USA); *β*-asarone was purchased from NIFDC (Beijing, China, 112018-201601), and the purity value is 96.8%; icarrin used was purchased from NIFDC (Beijing, China, 110737-201516), and the purity value is 94.2%; donepezil was obtained from the First Affiliated Hospital of Jinan University (Guangzhou, China); A3 was purchased from Santa Cruz Biotechnology (Santa Cruz, CA, USA); CSA was purchased from Selleck (Houston, Texas, USA). 3MA, high-glucose DMEM, FBS, trypsin, and PBS were obtained from Gibco (Gaithersburg, MD, USA); Cell Counting Kit-8 (CCK-8), Cytotoxicity LDH Assay Kit—WST (LDH), and Cellular Senescence Detection Kit—SPiDER-bGal were obtained from Dojindo Molecular Technologies, Inc. (Tokyo, Japan); the bicinchoninic acid (BCA) protein assay kit, Immunol Fluorescence Staining Kit, and Immunohistochemistry Staining Kit were obtained from Beyotime (Shanghai, China); anti-Beclin-1 antibody (EPR19662) (1 : 100, catalog no. ab207612, Abcam), anti-BACE1 antibody (EPR19523) (1 : 50, catalog no. ab183612, Abcam), anti-Synapsin I antibody-Synaptic Marker (1 : 800, catalog no. ab64581, Abcam), anti-SQSTM1/p62 antibody (1 : 800, catalog no. ab91526, Abcam), anti-Presenilin 1/PS-1 antibody (EP2000Y) (1 : 100, catalog no. ab76083, Abcam), anti-APPL antibody (1 : 500, catalog no. ab180140, Abcam), anti-PINK1 antibody (EPR20730) (catalog no. ab216144, Abcam), anti-Parkin antibody (PRK8) (1 : 100, catalog no. ab77924, Abcam), and the PC12 cells were obtained from the Institute of Cytobiology, Chinese Academy of Sciences.

### 2.2. Cell Culture and Handing

A*β*1-42 is the component found in amyloid plaques, and it has 42 amino acids. We used gradient concentrations of A*β*1-42 (0, 2.215, 4.43, 8.86, and 17.72 *μ*M) induced PC12 cell for 6, 12, 24, and 48 h. Cellular viability and toxicity were detected by CCK-8 and LDH assays, and the final condition we set was 7 *μ*M A*β*1-42 interference for 12 h.

### 2.3. Cell Proliferation and Cytotoxicity

Cell viability was detected using CCK-8 assay. For the CCK-8 assay, PC12 cells were seeded in 96-well plates and were cultured for 24 h. The cells were then treated with CCK-8 reagent and further cultured for 2 h according to the manufacturer's instructions. The absorbances were measured at 450 nm. Each group of experiments included 6 replicates and repeated three times. Cell injury was assessed through measuring the LDH activity in the supernatant of PC12 cells using an LDH kit according to the manufacturer's protocol.

### 2.4. Electron Microscopy

Electron microscopy was used to observe the autophagosome. For mouse hippocampus, the tissue was cleaned with PBS once and fixed for 24 h at 4°C in 2.5% glutaraldehyde and another 2 h in 1% osmium tetroxide. Then, the cells were made into ultrathin electron microscope sections. The cells were counted and adjusted to a density of 1 × 106 cells/ml and then fixed for 24 h at 4°C in 2.5% glutaraldehyde and another 2 h in 1% osmium tetroxide.

### 2.5. MSD Assay Analysis of A*β*38, A*β*40, and A*β*42

The analytical performance of the measurement of A*β*38, A*β*40, and A*β*42 levels in serum and in the hippocampal and prefrontal cortex tissues by MSD assay was assessed in terms of parallelism, sensitivity, accuracy, precision, lot-to-lot variability, selectivity, and freeze/thaw stability according to published guidelines (Meso QuickPlex SQ 120).

### 2.6. Flow Cytometric Evaluation of MMP and Ca^2+^

PC12 cells were cultured in DMEM as described above, seeded (2.4 × 10^5^ cells/mL, 200 *μ*L) on a *μ*-slide 8-well plate (ibidi) and cultured at 37°C in a 5% CO2 incubator overnight. The medium was removed, treatments were added to each well, and then, the cells were cultured at 37°C for 90 minutes in the 5% CO2 incubator. After the supernatant (100 *μ*L) was removed, JC-1 working solution (4 *μ*mol/L, 100 *μ*L) was added, and then, the cells were cultured at 37°C for 30 minutes in the 5% CO_2_ incubator.

Then, the cells were washed twice with 200 *μ*L of HBSS. Imaging buffer solution (200 *μ*L) was added, and the cells were observed under a laser confocal microscope (green: 488 nm (Ex), 500–550 nm (Em) and red: 561 nm (Ex), 560–610 nm (Em)) and flow cytometer (green: 488 nm (Ex), 515–545 nm (Em) and red: 488 nm (Ex), 564–604 nm (Em)).

### 2.7. Immunofluorescence (IF) Analysis

Briefly, approximately 9–10 slices from each tissue were incubated in blocking buffer (3% H2O2) for 10 min at room temperature. PC12 cells were cultured on the microscope slide at a density of 5 × 10^5^ cells/cm^2^ and were treated as described above. Then, endogenous peroxidase activity was quenched for 10 min in PBS containing 3% H2O2, chilled in water, and then immersed for 5 min in PBS. Thereafter, samples were incubated overnight with the primary antibody at 4°C and then incubated with the appropriate fluorescent probe-conjugated secondary antibodies for 1 h at room temperature while protected from light. Then, set the slides with SABC-FITC (diluted 1 : 200), and nuclei were stained with 4,6-diamidino-2-phenylindole (DAPI) at a 1 : 5,000 dilution. Then, the slices were mounted with an antifluorescent quencher. Pictures were taken with an Olympus microscope (BX53). Specific primary antibodies used 1 : 100. Plaque numbers per ROI were counted and quantified with image J. The protein was dyed green with SABC, and the nucleus was dyed blue with DAPI. The image merged the nuclear of the same cell that was marker and DAPI.

### 2.8. Western Bolt Analysis

The hippocampus and prefrontal cortex of mice were obtained from APP/PS1 brain tissue. Following electrophoresis, gels were transferred to a polyvinylidene difluoride (PVDF) membrane (Merck Millipore Ltd., Ireland) at 200 mA for 60 min. The membrane was incubated for 10 min in Tris-Buffered Saline Tween-20 (TBST) buffer containing 5% skimmed milk on the shaker. Antibodies used at a 1 : 1,000 dilution. Secondary antibodies used at a 1 : 3,000 dilution. All the molecular weights labelled were calculated by the size of the protein of the ladder/marker (actin).

### 2.9. DALGreen Autophagy Detection and Mitophagy Detection

PC12 cells were incubated at 37°C for 30 minutes with 250 *μ*l of 1 *μ*mol/l DALGreen working solution. After the cells were washed with the culture medium twice, the culture medium or amino acid-free medium (Wako Pure Chemical Industries, Ltd., Code: 048-33575) was added to the well. After 6-hour incubation, the cells were washed with Hanks' HEPES buffer twice and then DALGreen was observed by confocal fluorescence microscopy. Finally, the cells were observed by confocal fluorescence microscopy (Ex: 561 nm, Em: 650 nm) (lysosomes, Ex: 488 nm, Em: 502-554 nm).

### 2.10. Mouse Behavioural Tests

Mice were trained in the water maze with four trials per day for 5 days. They were placed in a pool of water containing a platform just below the surface of the water. They escaped from the maze (i.e., they were removed from the pool) when they found the platform. Distal visual cues were arrayed around the room, and in general, the mice were able to learn the location of the hidden platform based on these cues. Water maze performance was analysed by the amount of time spent in the target quadrant (sec). When a mouse exceeded 300 s, it was recorded as 300 s.

All mice were allowed one day of rest after the water maze test and the passive avoidance test before the step-down test was conducted using a step-down recorder (Jinan Yiyan Technology Development Co., Ltd.) and a previously described method [22].

### 2.11. Statistical Analysis

All data were expressed as means ± standard deviation. Differences between two groups were analysed using the *t*-test, and differences among three or more groups were analysed using single-factor analysis of variance (one-way ANOVA) with SPSS (version 16.0 for Windows). Differences were considered statistically significant at *P* < 0.05.

## 3. Result

### 3.1. The Composition of Icariin Combined with *β*-Asarone Was Determined by ^13^C-NMR

**Figure 1:**
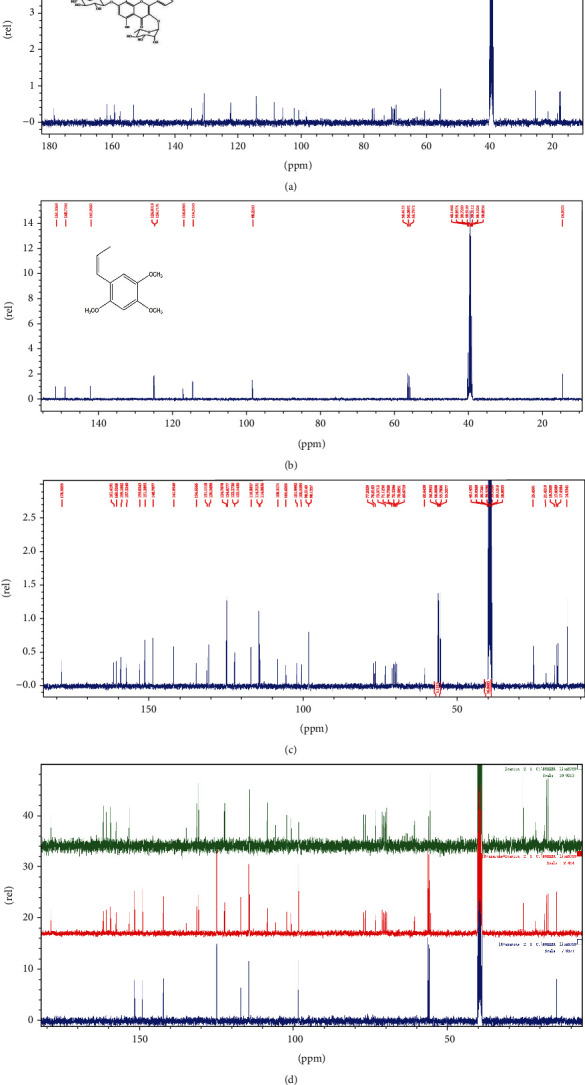
^13^C NMRCS of ICA, *β*-asarone, and combined application. (a) Icarrin, ICA, (b) *β*-sarone, and (c) ICA+*β*-asarone. (d) The three groups of spectra are superimposed and combined, and all peaks and peak shapes are benchmarked after combination.

^13^C-NMR spectrum is shown in Figure 1, which clearly shows the residual dissolution peak containing the dissolution of CDCl3. There were three groups: single icarrin, single *β*-asarone, and ICA+*β*-asarone, as can be seen from the detection of icariin in Figure 1(a). 37 different ^13^C chemical shifts were presented in this spectrum, as can be seen from the detection of icariin in Figure 1(b). For *β*-asarone, 19 different ^13^C chemical shifts were presented in this spectrum. From the results of icariin+*β*-asarone in Figure 1(c), it can be seen that 45 different ^13^C chemical shifts are presented in the spectrum. The number of ^13^C above is consistent with the sum of the two monomers and CDCl3, and the spectral peaks are clearly separated. Figures 1(a) and 1(b) are splicing according to the chemical shift, and it can be seen that no new ^13^C structure is formed after the mixing of the two. This also provides a certain pharmacological basis for the follow-up experiments.

### 3.2. Defective Mitochondrial Function and Mitophagy in the APP/PS1 Mouse and in the PC12 Cell Model

**Figure 2:**
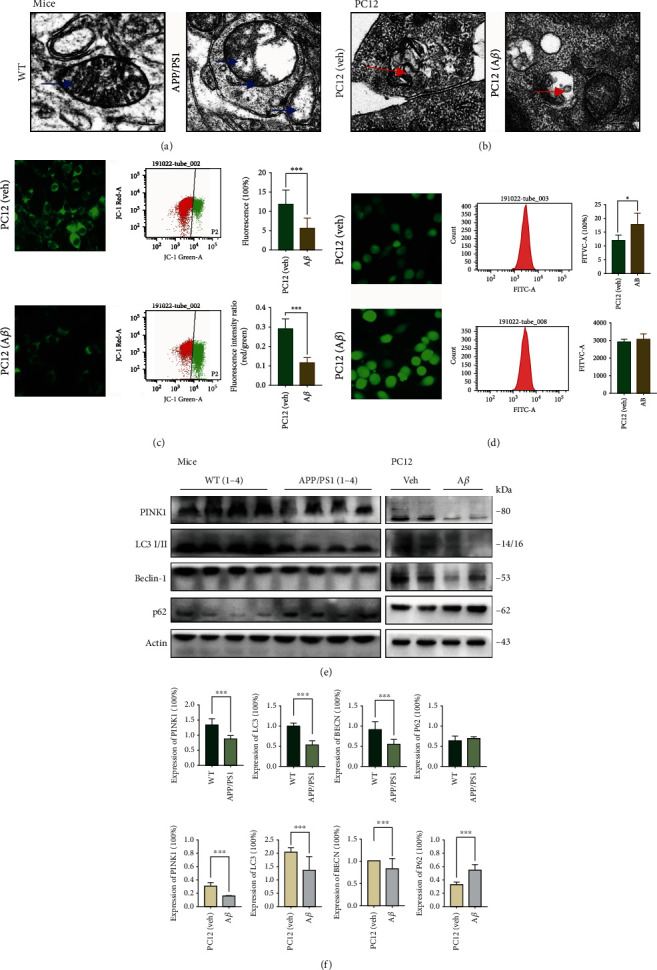
Mitochondrial dysfunction and defective mitophagy in APP/PS1 mouse hippocampus and A*β*1-42-induced PC12 cell model. (a, b) Representative set of electron microscopy images (scale bar 1 *μ*m and magnification 23,000x). (c) Representative *ΔΨ*m detected with JC-1 Kit, used IF (scale bar 100 *μ*m and magnification 400x), and FCM (R1 : R2 represents the monomers to aggregates ratio). Compared with the PC12 group, the cell model displayed a significantly lower red/green ratio. (d) Representative intracellular Ca^2+^ intensity detected with Fluo-4 am, with IF (scale bar 100 *μ*m and magnification 400x) and FCM. The concentration of cytoplasmic free calcium was markedly higher in the APP/PS1group. (e) Representative Western blotting bands using total protein extracts from the hippocampus and PC12 cells (MW: molecular weight). (f) Quantification of protein expression levels between control and AD groups. For APP/PS1 mouse, the level of PINK1, LC3 I/II, and Beclin-1 decreased while p62 increased. For PC12 cells, the level of PINK1, LC3 I/II, and Beclin-1 decreased while p62 increased. (b–f) All the center values represent means, and the error bars represent SD. Compared with the control group, ^∗^*P* < 0.05 and ^∗∗∗^*P* < 0.001; one-away ANOVA. At least three experiments were repeated independently with similar results. Full scans of all the blots are in the Supplementary Note (available here).

To uncover the cellular and molecular causes of mitochondrial impairment and the accumulation of damaged mitochondria in AD, we examined the changes in mitochondria in the hippocampus of APP/PS1 mice and in the PC12 cell model. The hippocampal neurons in the APP/PS1 samples displayed altered mitochondrial morphology characterized by reduced size and excessive mitochondrial damage compared to those in the healthy control samples (Figure 2(a)). The cell model also showed the same changes, including swollen and deformed mitochondria (Figure 2(b)). *ΔΨ*m is not only used for ATP synthesis but is also a factor that determines the viability of mitochondria participating in the elimination of disabled mitochondria [23]. In PC12 cells, we detected a decrease in mitochondrial viability in the model group from both flow cytometric detection and laser confocal microscopy. JC-1, a cationic carbocyanine dye (green), exhibits potential-dependent accumulation in mitochondria where it forms J-aggregates (red), whereas if it remains as monomer, it shows green fluorescence. The concentration was reduced, the monomer emitted more green fluorescence in the AD group, and the *ΔΨ*m remained at a lower level (Figure 2(c)). Accurate measurements of intracellular Ca^2+^ concentration allow for a more comprehensive understanding of Ca^2+^-regulated cell functions and signalling pathways.

Fluo-4 is a calcium-specific fluorescent dye that exhibits a greater than 100-fold increase in fluorescence upon binding Ca^2+^ ions. The AD cells showed increased intracellular calcium levels compared to those of the control cells (Figure 2(d)). Combined with the results of our previously published articles, we have demonstrated that autophagy is inhibited in these AD models, but we also need to systematically examine the protein levels of major factors involved in mitochondrial metabolism and mitophagy. The expression levels of the mitochondrial quality control protein PINK1 and the autophagy proteins LC3 I/II and Beclin-1 were decreased in both the hippocampal and PC12 cell samples. p62, a substrate degraded by autophagy, showed the opposite tendency (Figures 2(e) and 2(f)). These findings showed the accumulation of damaged mitochondria in the hippocampus of APP/PS1 mice and the PC12 cell model accompanied by defects in autophagy and mitophagy.

### 3.3. The Combination Treatment Ameliorates Cognitive Decline and AD Pathology in APP/PS1 Mice

In the part of pharmacodynamic, testing was divided into 6 groups, respectively, normal WT mice, APP/PS1, single icarrin, single *β*-asarone, ICA+*β*-asarone, and donepezil. Based on the relationship between the effective dose and the dose used in the animal experiments, we chose to treat the mice in the combination group with 15 mg/kg/d *β*-asarone and 20 mg/kg/d icariin.

**Figure 3:**
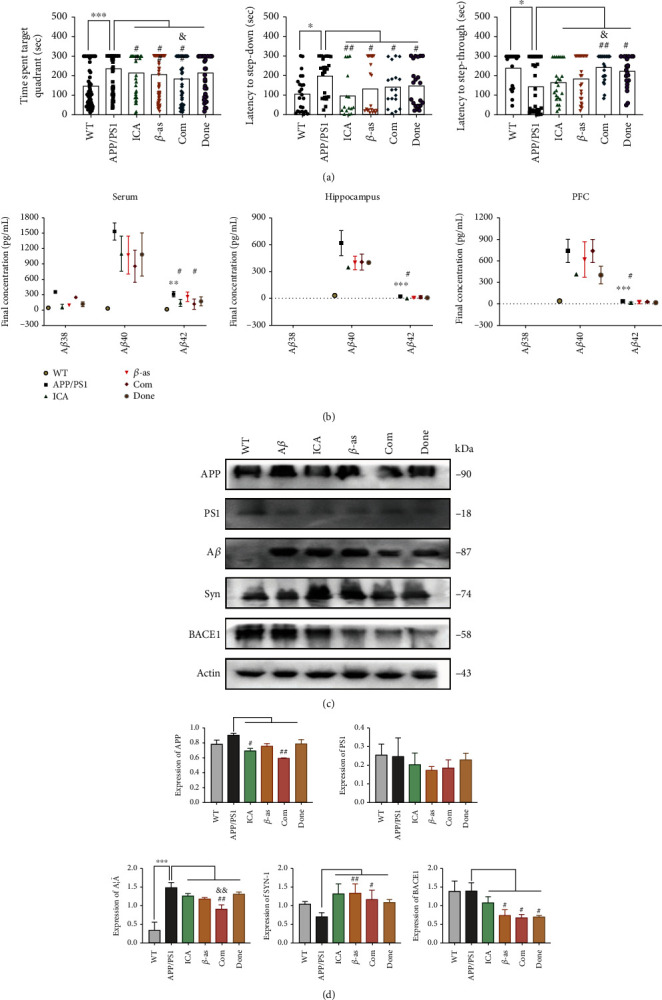
Combination drug ameliorates cognitive decline and AD pathology in APP/PS1 mouse. APP/PS1 mice were treated as groups by daily gavage for 4 weeks starting from 3 months of age; then, behavioural tests were performed, and brains and serum were subjected to histological and molecular analyses. The groups include Ctrl (control), AD (APP/PS1 or PC12 cell model), ICA (icariin), *β*-as (*β*-asarone), Com (the combination of *β*-asarone and icariin), and DONE (donepezil). (a) Morris water maze, step-down, and step-through were tested after administration (each group, days = 6, *n* = 12, and total = 72). Time spent target quadrant was decreased after administration, but the Com showed better effect. (b) The analytical performance of the MSD A*β*38, A*β*40, and A*β*42 in serum, hippocampal, and prefrontal cortex tissue assays in terms of parallelism, sensitivity, accuracy, precision, lot-to-lot variability, selectivity, and freeze/thaw stability was assessed according to published guidelines (*n* = 3 per group). A*β*38 in mouse hippocampus and PFC was not detected, and A*β*40 and A*β*42 revealed a significant trend in AD groups. Combination drug can inhibit the expression of A*β*40 and A*β*42. (c) APP, PS1, A*β*, BACE1, and Syn levels in hippocampal tissues with WB. (d) Representative quantification of protein expression levels between different groups. Compared with the control group, ^∗^*P* < 0.05, ^∗∗^*P* < 0.01, and ^∗∗∗^*P* < 0.001; compared with the AD group, ^#^*P* < 0.05 and ^##^*P* < 0.01; compared with *β*-asarone or icarrin, ^&^*P* < 0.05 and ^&&^*P* < 0.01; one-away ANOVA. At least three experiments were repeated independently with similar results. Full scans of all the blots are in the Supplementary Note.

To investigate the effect of the combination treatment on AD, we tested the behaviour of APP/PS1 mice (Figure 3(a)) and analysed the levels of AD-related proteins (Figure 3(b)–3(d)). The transgenic AD mice exhibited a longer swimming time (*P* < 0.001) and a greater number of errors (*P* < 0.01) in the water maze test and longer latency in the step-down and step-through tests (*P* < 0.05). Treatment with either *β*-asarone, icariin, combined *β*-asarone and icariin, or donepezil all improved learning and memory retention in the AD mice, and the combination treatment showed a better effect than the individual treatments (*P* < 0.05) (Figure 3(a)). The MSD assay was used to test the three proteins A*β*38/A*β*40/A*β*42 in the serum, hippocampus, and PFC; from the data in Figure 2(b), it can be seen that A*β*38 was only detected in the serum, while A*β*38 was not detected in hippocampal and PFC tissues. The overall expression of A*β*40 was higher than that of A*β*42, which is consistent with the existing knowledge that in human cerebrospinal fluid and blood, A*β*40 expression is 10 times and 1.5 times higher than that of A*β*42, respectively; however, A*β*42 is more toxic and more likely to aggregate, thereby forming a toxic precipitate and triggering neurotoxicity, so A*β*42 is the main focus among the three similar proteins in AD research. The results of the simultaneous comparison of the three proteins A*β*38, A*β*40, and A*β*42 suggest that the expression analysis of A*β*42 is more meaningful for AD than those of A*β*38 and A*β*40. The analysis of A*β*42 is shown separately. In the normal control mice, a small amount of A*β*42 was detected in only the serum, whereas no expression of A*β*42 was detected in the hippocampus or PFC. The contents of A*β*42 in the serum, hippocampus, and PFC in the AD group were significantly higher than those in the normal group (*P* < 0.01 and *P* < 0.001), and the contents of A*β*42 in the three tissues decreased after the application of icariin or the combination treatment (*P* < 0.05). Western blotting was used to simultaneously detect APP, PS1, A*β*, Syn, and BACE1.

The combination treatment did not change the expression level of PS1 but decreased the levels of APP, A*β*, Syn, and BACE1. This result demonstrated that the combination therapy effectively attenuated the expression of AD-related pathological metabolites and toxic proteins.

Furthermore, the expression of synaptophysin was most pronounced in the combination group (Figure 3(c)). These results indicate that the combination treatment ameliorated memory deficits in the APP/PS1 mouse model, promoted the degradation of toxic proteins, and increased the expression of synaptic proteins.

### 3.4. The Combination Treatment Inhibits Cell Damage in the A*β*1-42 PC12 Cell Model of AD

**Figure 4:**
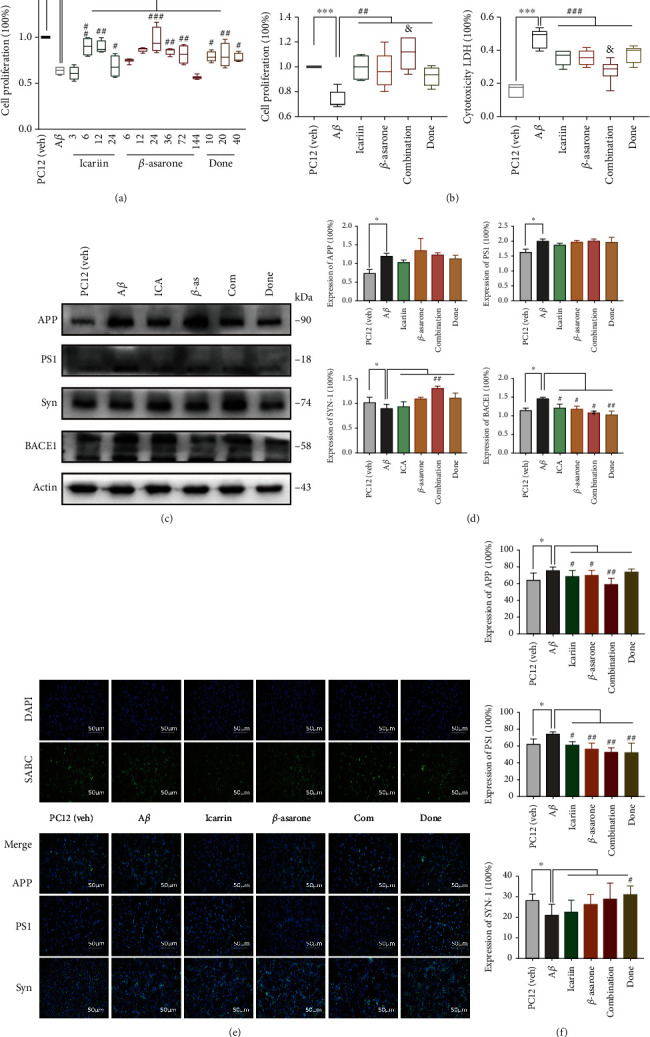
Combination drug inhibited cell damage and toxic protein deposition in A*β*1-42 PC12 cell model of AD. (a) PC12 cells were treated with 7 *μ*M A*β*1-42 for 12 h; then, we cultured the model cells with icariin, *β*-asarone, combination drug, or donepezil for another 12 h. (b) Cell proliferation and cytotoxicity LDH were detected as the above groups. (c) APP, PS1, BACE1, and Syn levels in PC12 cells with WB. (d) Quantification of protein expression levels in cells. At least three experiments were repeated independently with similar results. Full scans of all the blots are in the Supplementary Note. (e) IF assay of APP, PS1, and Syn was detected after the treatment (scale bar 100 *μ*m and magnification 200x). SABC-DyLight marker protein expression, DAPI marker cell nucleus, also with a merge paper. Scale bar 100 *μ*m. (f) Quantification of protein expression levels.

We cultured PC12 cells in vitro and found that 7 *μ*M A*β*1-42 for 12 h was the optimal A*β*1-42 concentration for this model. In the initial experiments, we investigated the effects of gradient concentrations of *β*-asarone (12, 24, 36, 72, and 144 *μ*M) or icariin (3, 6, 12, and 24 *μ*M). We found that the protective effect of *β*-asarone and icariin on cell proliferation was dose-dependent; when the *β*-asarone/icariin concentrations were increased to 72/12 *μ*M, the proliferation capacity of the PC12 cells started to decrease. At the *β*-asarone/icariin concentrations of 144/24 *μ*M, viability was further reduced (Figure 4(a)). We chose 36 *μ*M *β*-asarone and 6 *μ*M icariin as the working concentrations. The concentration of donepezil was also calculated according to the effective doses in humans and in cells, and the final concentration was determined by means of a preliminary experiment.

After we established a stable AD cell model, we investigated the effects of 6 *μ*M icariin, 36 *μ*M *β*-asarone, the combination of 36 *μ*M *β*-asarone and 6 *μ*M icariin, and 10 *μ*M donepezil. Compared with the individual icariin and *β*-asarone treatments, the combination treatment improved cell proliferation and decreased cell damage (Figure 4(b)). We also analysed APP, PS1, Syn, and BACE1 by Western blotting (Figures 4(c) and 4(d)). The combination treatment suppressed the levels of APP and PS1 and increased the expression of Syn. Furthermore, we observed the expression of APP, PS1, and Syn with IF (Figures 4(e) and 4(f)). Collectively, these data suggested that the combination treatment showed a better effect in inhibiting the accumulation of toxic proteins and metabolites and promoting the secretion of synaptophysin, thus exerting a protective effect in AD pathology.

Icariin, *β*-asarone, and the Com promote clear APP and PS1, combination group showed better effect in APP. Donepezil has a stronger effect on PS1 and Syn. The experiments were repeated twice independently with similar results. Compared with the control group, ^∗^*P* < 0.05 and ^∗∗∗^*P* < 0.001; compared with the AD group, ^#^*P* < 0.05, ^##^*P* < 0.01, and ^###^*P* < 0.01; compared with *β*-asarone or icarrin, ^&^*P* < 0.05; one-away ANOVA.

### 3.5. The Combination Treatment Activated Autophagy and Mitophagy

**Figure 5:**
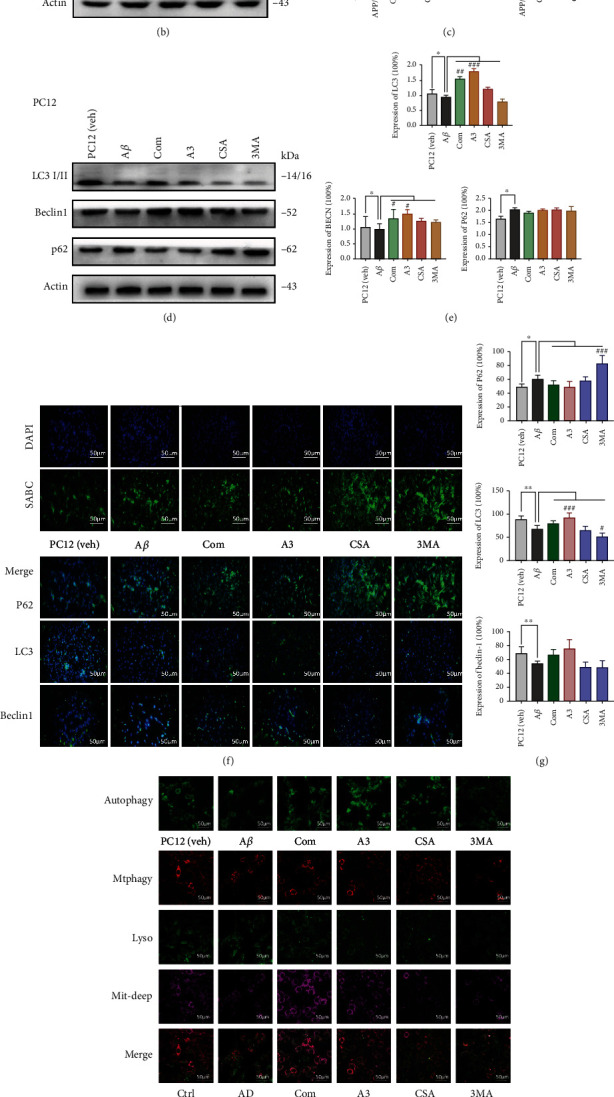
Combination drug intact autophagy and mitophagy. (a–c) APP/PS1 mouse. (a) Representative set of electron microscopy images showing autophagic vacuoles-autophagosomes (AU) and multilamellar bodies (MLB) (arrows) (scale bar 1 *μ*m and magnification 23,000x). (b) Representative Beclin-1, LC3, p62, PINK1, Parkin, and p-Parkin levels with WB and (c) showed quantification of protein expression levels. (d–i) PC12 cells. (d) Beclin-1, LC3, and p62 levels with WB and (e) quantification of protein expression levels. (f) IF assay of Beclin-1, LC3, and p62 was detected after the treatment (scale bar 100 *μ*m and magnification 400x). SABC-DyLight marker protein expression, DAPI marker cell nucleus, also with a merge paper and (g) showed quantification of protein expression levels. (h, autophagy) Fluorescence images of autophagic cells. DALGreen was utilized for the imaging of autophagic cells. Starved cells were observed in the culture medium and (i) the data quantification (scale bar 100 *μ*m and magnification 630x) (Ex: 488 nm, Em: 500-563 nm). (h, Mtphagy) Fluorescence images of mitophagy cells with mitophagy staining (scale bar 100 *μ*m and magnification 630x) (Ex: 561 nm, Em: 650 nm). Lysosomes, Ex: 488 nm, Em: 502-554 nm. Mitochondrial staining with Mitobright Deep Red, Ex: 640 nm, Em: 656-700 nm. (i) Quantification of the data. Compared with the control group, ^∗^*P* < 0.05, ^∗∗^*P* < 0.01, and ^∗∗∗^*P* < 0.001; compared with the AD group, ^#^*P* < 0.05, ^##^*P* < 0.01, and ^###^*P* < 0.001; one-away ANOVA. At least three experiments were repeated independently with similar results. Full scans of all the blots are in the Supplementary Note.

There is substantial evidence that dysregulation of autophagy occurs in both AD animal models and AD patients [24]. To determine the occurrence of autophagy, we performed electron microscopy on hippocampal slices of APP/PS1 mice. In this part, we divided into 6 groups, respectively, normal WT mice/PC12 (veh), APP/PS1/PC12 (A*β*), ICA+*β*-asarone, A3, and CSA. Electron microscopy found that the combination treatment promoted the formation of autophagic vacuoles (autophagosomes, AU) and multilamellar bodies (MLB) and increased the density of synapses (Figure 5(a)). Mitophagy defects lead to the accumulation of damaged mitochondria, energy deprivation, and eventually neuronal loss. Hence, compromised mitophagy could contribute substantially to AD pathology and cognitive decline. PINK1 triggers signals for mitophagy initiation and Parkin-mediated signal amplification. When we detected the expression of Beclin-1, LC3 I/II, p62, PINK1, and Parkin in the hippocampus of APP/PS1 mice, we clearly found that the combination treatment induced autophagy and mitophagy (Figures 5(b) and 5(c)). Then, Western blotting and IF were used to measure the above proteins in PC12 cell models (Figures 5(d)–5(g)). Moreover, we used DALGreen to measure autophagy (Figures 5(h) and 5(i)). DALGreen is a small fluorescent molecule with unique properties that emits fluorescence under hydrophobic and acidic conditions and can detect autolysosomes. The combination and A3 groups displayed a decreased amount of p62, while the other proteins tended to increase (Figures 5(f) and 5(g)). Taken together with the DALGreen findings, all the results indicated that the combination treatment significantly promoted the occurrence of autophagy (Figure 5(h), autophagy).

Furthermore, we detected mitophagy, mitochondria, and lysosomes with Mtphagy Dye (Figure 5(h), Mtphagy). The combination drug led to an increase in the fluorescence intensities of the Mtphagy, Mitochondria Deep Bright, and Lysosome dyes. This indicates that the combination treatment promoted mitochondrial autophagy while improving mitochondrial function. However, A3 promoted mitophagy but had little effect on mitochondria (Figure 5(h), Mtphagy). Thus, the combination treatment acts as a specific autophagy/mitophagy activator and has a certain protective effect against the destruction of mitochondrial function in the AD state.

### 3.6. Mitophagy Induced by the Combination Treatment Inhibits Amyloid-*β* and Reverses Cognitive Deficits

**Figure 6:**
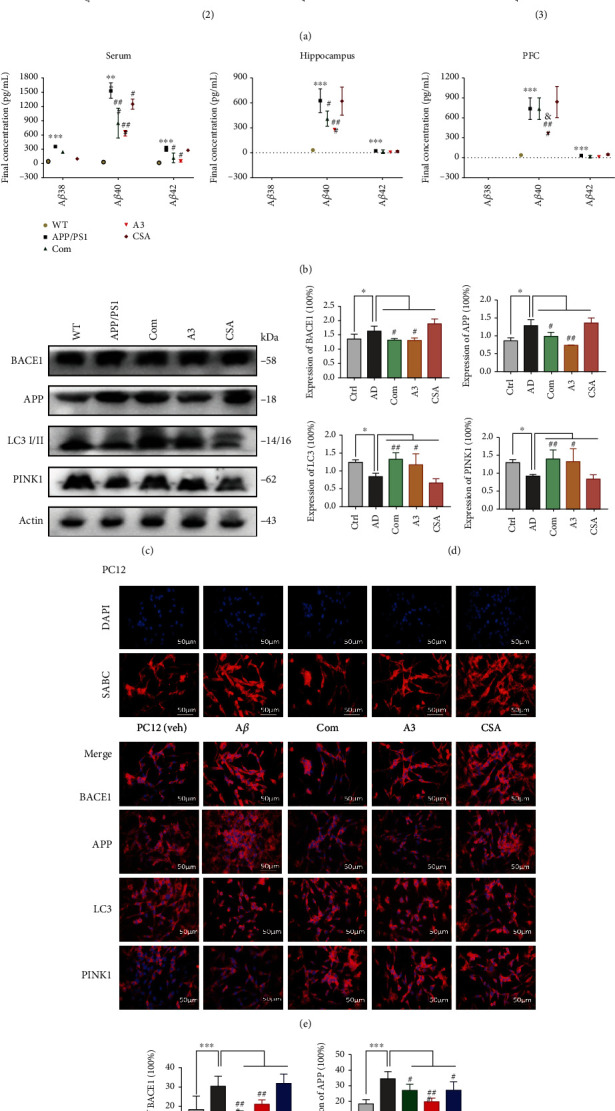
Mitophagy induces by combination drug inhibits amyloid-*β* and reverses cognitive deficits. APP/PS1 mice were treated as groups by daily gavage for 4 weeks, behavioural tests were performed, and brains and serum were subjected to histological and molecular analyses. (a) Morris water maze, step-down, and step-through were tested after administration (each group, days = 6, *n* = 4‐12, and total = 24‐72). Water maze performance was analysed by the amount of time spent in the target quadrant (sec). APP/PS1 mice showed significant memory loss in all three tests. Time spent target quadrant was decreased after the combination and A3. (b) The analytical performance of the MSD A*β*38, A*β*40, and A*β*42 in serum, hippocampal, and prefrontal cortex tissue assays in terms of parallelism, sensitivity, accuracy, precision, lot-to-lot variability, selectivity, and freeze/thaw stability was assessed according to published guidelines (*n* = 3 per group). A*β*38 in mouse hippocampus and PFC was not detected, and A*β*40 and A*β*42 revealed a significant trend in AD groups. Autophagy activator A3 drug can inhibit the expression of A*β*40 and A*β*42. (c) BACE1, APP, LC3 I/II, and PINK1 levels in hippocampal tissues with WB. (d) Quantification of protein expression levels between each group. (e) IF assay of BACE1, APP, LC3 I/II, and PINK1 was detected after the treatment (scale bar 100 *μ*m and magnification 400x) in PC12 cells. Cy3-DyLight marker protein expression, DAPI marker cell nucleus, also with a merge paper. Scale bar 100 *μ*m. (f) Quantification of protein expression levels in cells. The experiments were repeated twice independently with similar results. Compared with the control group, ^∗^*P* < 0.05, ^∗∗^*P* < 0.01, and ^∗∗∗^*P* < 0.001; compared with AD, ^#^*P* < 0.05, ^##^*P* < 0.01, and ^###^*P* < 0.001; one-away ANOVA.

To obtain a systematic, unbiased overview of how this combination treatment affects memory-related neuronal functions in AD, we performed further exploration into to the relationship between the combination treatment, autophagy/mitophagy, and AD. We performed behavioural tests after treatment with the mitophagy-activating agent A3 and the mitophagy-inhibiting agent CSA. The AD mice showed a significant decrease in behavioural indicators compared with those of the control mice (Figure 6(a)). In the water maze test, the time spent in the target quadrant in the combination treatment and A3 groups was reduced compared with that in the AD group. Moreover, the amount of time the CSA group spent in the target quadrant was extended. The results of the platform test were consistent with those of the water maze. In the step-through test, only the combination treatment group exhibited reduced time, while there were no obvious changes in the other groups. We also used the MSD assay to detect the proteins A*β*38, A*β*40, and A*β*42 in the serum, hippocampus, and PFC (Figure 6(b)). As shown in Figure 5(b), A*β*38 was only detected in the serum, while hippocampal and PFC tissues expressed lower levels of A*β*38. In the normal control mice, only a small amount of A*β*42 was detected in the serum, while A*β*42 expression was not detected in the hippocampus or PFC. The content of A*β*42 in the serum, hippocampus, and PFC in the AD group was significantly higher than that in the normal group (*P* < 0.05 and *P* < 0.001). After icariin was applied in combination with *β*-asarone or activator A3, the contents of A*β*40 and A*β*42 were significantly reduced. The ability of the activator treatment to inhibit A*β*40 in the prefrontal cortex was better than that of the combination treatment (*P* < 0.05). Next, we examined the changes in AD-related indicators in the hippocampus of the above groups. BACE1 is the putatively rate-limiting initiating enzyme in A*β* generation, and it is considered a prime drug target for lowering cerebral A*β* levels in the treatment and/or prevention of AD [24]. LC3 is a central protein in the autophagy pathway that functions in autophagy substrate selection and autophagosome biogenesis. PINK1 and Parkin are established mediators of mitophagy, the selective removal of damaged mitochondria by autophagy [25]. We found that the combination treatment led to decreased expression of APP and BACE1, while LC3 I/II and PINK1 expression increased. Moreover, A3 treatment promoted the levels of LC3 I/II and PINK1, and the levels of APP and BACE1 were both suppressed. Western blot experiments showed the same results (Figures 6(c) and 6(d)). Then, we also used the cell model to analyse the levels of the above proteins in the same groups. The results were basically consistent with those from the APP/PS1 mice. The combination treatment and the A3 treatment promoted the expression of LC3 I/II, and PINK1, thus inducing autophagy/mitophagy and inhibiting BACE1 and APP (Figures 6(e) and 6(f)). We can conclude from the above results that the combination treatment reduced the deposition of toxic proteins by promoting autophagy/mitophagy.

## 4. Discussion

In the current study, we describe a combination treatment of icariin and *β*-asarone with efficacy in a cross-species model of AD. This is the first combination of these two monomers in AD research. We divided the research into three parts: (1) the effect of the combination treatment on AD models, (2) the effect of the combination treatment on autophagy/mitophagy, and (3) the effect of the combination treatment on AD by affecting autophagy/mitophagy on PINK1/Parkin. Through the above analysis, we aimed to systematically examine the effect and mechanism of combined icariin and *β*-asarone treatment on AD. Based on our previous years of research, we obtained the optimal concentration of the two monomers and combined the two active ingredients to generate a stable, single-dose form. The combination of *β*-asarone and icariin showed a better effect in inhibiting the accumulation of toxic proteins and metabolites in vivo and in vitro, and we found that the expression of BACE1 was significantly inhibited. The therapeutic potential of BACE1 inhibition has been investigated in the last decade. However, even though BACE1 inhibitors efficiently lower brain A*β* levels, clinical trials still have not demonstrated improvements in cognitive function when BACE1 inhibitors are administered to mild-to-moderate AD patients, calling into question the real worth of these potential anti-AD drugs and the designs of the clinical trials [26].

Therefore, in addition to the basic pathological changes, we also examined the effect of the combination on autophagy/mitophagy. We identified the changes in both in vivo and in vitro models. We compared the combination treatment with mitophagy activator and mitophagy inhibitor treatments, and the experimental results suggested that the combination treatment effectively promoted the occurrence of autophagy and protected against mitochondrial damage. Furthermore, promoting autophagy also exerted a protective effect by clearing metabolic products and promoting the secretion of synaptophysin, thus exerting a protective effect in AD pathology.

AD is a heterogeneous disease with a complex pathobiology. Approximately 40 million people over the age of 60 suffer from AD worldwide, and the number of patients is increasing and doubles every 20 years [27, 28]. Several recent fundamental discoveries have highlighted important pathological roles of other critical cellular and molecular processes. However, the deposition of extracellular *β*-amyloid protein as neuritic plaques and the intracellular accumulation of hyperphosphorylated tau as neurofibrillary tangles remain the most important neuropathological criteria for AD diagnosis [29]. At present, clinical drug treatments are mainly divided into two categories: acetylcholinesterase inhibitors (AChEIs) and antagonists of the N-methyl-D-aspartic acid (NMDA) receptor. As neurotransmitter regulators, these drugs can relieve symptoms for a short time but cannot delay the progression of AD [30]. Recently, a new drug, aducanumab, has been sent to the FDA after analysis yielded positive results in early Alzheimer's disease [31]. Donepezil is a representative cholinesterase inhibitor that improves the ability of impaired nerve endings to transmit messages between nerve cells [32]. These drugs are based on a single compound with a single target. There are also various new descriptive hypotheses regarding the causes of AD, including the mitochondrial cascade hypothesis, the calcium homeostasis hypothesis, the neurovascular hypothesis, the inflammatory hypothesis, the metal ion hypothesis, and the lymphatic system hypothesis. However, the ultimate aetiology of AD remains obscure. Among the clinical trials in which each of the various hypotheses for AD were tested up to the year 2019, the amyloid hypothesis was the most heavily tested (22.3% of trials), followed by the neurotransmitter hypothesis (19.0% of trials)^41^. Autophagy dysfunction followed by metabolic or oxidative stress as a result of aging leads to the accumulation of indigestible or incompletely degraded materials containing the components for A*β* generation, including APP and its processing enzymes [33, 34]. Some studies have also shown that promoting mitochondrial autophagy can ameliorate the cognitive impairment characteristic of both A*β* and tau pathologies [35].

The current data broaden our understanding of the effect of the combination of *β*-asarone and icariin, which promotes the clearance of toxic proteins such as APP, PS1, and BACE1, promotes the formation of autophagosomes, and increases the expression of Beclin-1, PINK1, and p/Parkin. These structural and functional enhancements help to explain the improved learning and reversed cognitive deficits observed after administration. Our team has long been committed to the study of neurodegenerative diseases. We believe that the pathogenesis of AD is very complicated, and it is possible that important links have not yet been revealed, maybe because the pathophysiological mechanism of AD is not clear, and the combination therapy improved the pathological results caused by a combination of various factors through different molecular mechanisms. Therefore, we should pay attention to multiple links and targets and try to explore the use of combined drugs, thus accelerating the study of recommending AD.

However, these results also raise many important questions, such as (1) if AD is a progressive disease, when is the best time for drug application? (2) Since mitophagy is also a dynamic and ongoing process, should the effectiveness of a treatment be evaluated based on the time at which it is given to the patient? While it is difficult to address these questions with only AD models, we are conducting more related clinical trials to further explain this problem.

## 5. Conclusion

The evidence reported herein strongly indicates that mitochondrial function and mitophagy may be defective in AD. The combination of *β*-asarone and icariin modulated mitophagy and improved the pathological damage of AD, potentially by improving the function of mitochondria and promoting the occurrence of mitophagy. Thus, targeting the regulation of autophagy at both the neuronal and organismal levels may be a promising therapeutic approach. New autophagy/mitophagy activator-based therapies might represent an innovative and attractive strategy in regenerative medicine.

## Data Availability

The data used to support the findings of this study are available from the following link: https://www.jianguoyun.com/p/DVrSVlUQgN7dCRj9-IYE.

## Supplementary Materials

Supplementary Materials Graphical abstract is included as supplementary file. In APP/PS1 mouse and A*β*1-42 in induced PC12 cell models, the amyloid plaques formed by BACE1 shearing APP impair mitochondrial function and autophagy. This results in impaired nerve function and increased metabolic burden in the brain. *β*-Asarone is the main constituents of *Acorus tatarinowii Schott* and plays important effects in diseases such as neurodegenerative and neurovascular diseases. Icariin (ICA) is a major active ingredient of Epimedium which attracts increasing attention because of its unique pharmacological effects in degenerative disease. The combination should inhibited cell and mitochondrial damage via induced autophagy/mitophagy. Therefore, the function of mitochondria and autophagy are repaired, the precipitation of toxic proteins is reduced, and the neurological function is recovered.
