# Supplementary material for: The Combination of β-Asarone and Icariin Inhibits Amyloid-β and Reverses Cognitive Deficits by Promoting Mitophagy in Models of Alzheimer's Disease
Source: Oxid Med Cell Longev. 2021 Nov 30;2021:7158444. doi: 10.1155/2021/7158444 (PMC8651403; doi:10.1155/2021/7158444)
Supplement: Supplementary Materials — Graphical abstract is included as supplementary file. In APP/PS1 mouse and Aβ1-42 in induced PC12 cell models, the amyloid plaques formed by BACE1 shearing APP impair mitochondrial function and autophagy. This results in impaired nerve function and increased metabolic burden in the brain. β-Asarone is the main constituents of Acorus tatarinowii Schott and plays important effects in diseases such as neurodegenerative and neurovascular diseases. Icariin (ICA) is a major active ingredient of Epimedium which attracts increasing attention because of its unique pharmacological effects in degenerative disease. The combination should inhibited cell and mitochondrial damage via induced autophagy/mitophagy. Therefore, the function of mitochondria and autophagy are repaired, the precipitation of toxic proteins is reduced, and the neurological function is recovered. [file 7158444.f1.pdf]

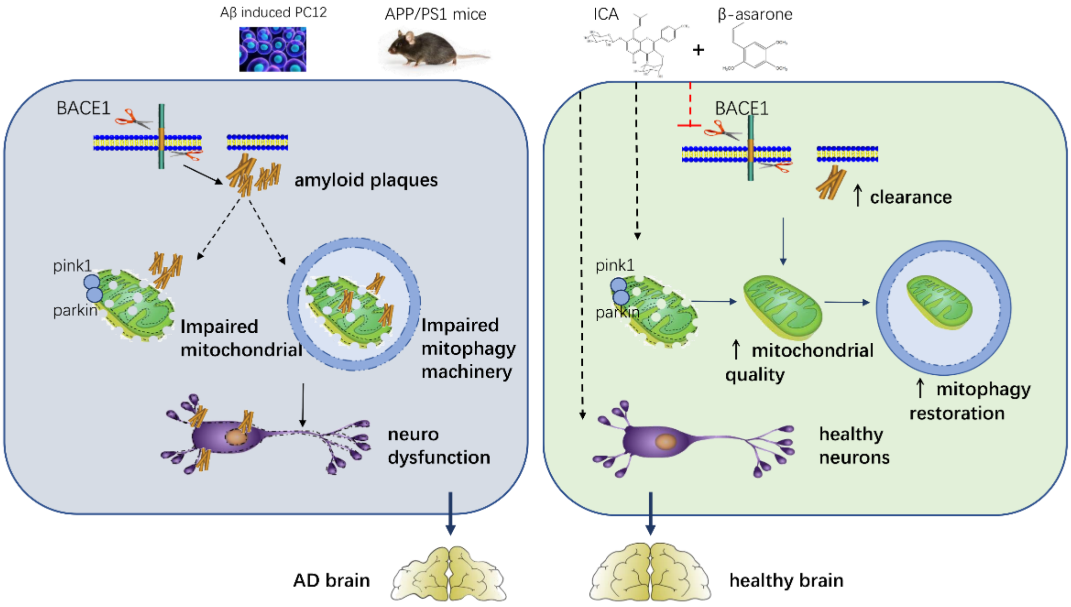

Graph abstract. In APP/PS1 mouse and $A\beta_{1-42}$ in induced PC12 cell models, The amyloid plaques formed by BACE1 shearing APP impair mitochondrial function and autophagy. This results in impaired nerve function and increased metabolic burden in the brain. β-Asarone is the main constituents of Acorus tatarinowii Schott and plays important effects in diseases such as neurodegenerative and neurovascular diseases. Icariin (ICA) is a major active ingredient of Epimedium which attracting increasing attention because of its unique pharmacological effects in degenerative disease. The combination should inhibited cell and mitochondrial damage via induced autophagy/mitophagy. Therefore, the function of mitochondria and autophagy are repaired, the precipitation of toxic proteins is reduced, and the neurological function is recovered.
